# The prevalence of childhood sexual experiences and intimate partner violence among transgender women in China: Risk factors for lifetime suicidal ideation

**DOI:** 10.3389/fpubh.2022.1037622

**Published:** 2023-01-12

**Authors:** Lulu Xu, Ruijie Chang, Yingjie Chen, Danni Xia, Chen Xu, Xiaoyue Yu, Hui Chen, Rongxi Wang, Yujie Liu, Shangbin Liu, Xin Ge, Tiecheng Ma, Yiwen Zhou, Ying Wang, Sunxiang Ma, Yong Cai

**Affiliations:** ^1^School of Public Health, School of Medicine, Shanghai Jiao Tong University, Shanghai, China; ^2^Shenyang Consultation Centre of AIDS Aid and Health Service, Shenyang, China; ^3^Department of Plastic and Reconstructive Surgery, Shanghai Ninth People's Hospital, Shanghai Jiao Tong University School of Medicine, Shanghai, China; ^4^Hongqiao International Institute of Medicine, Tongren Hospital, Shanghai Jiao Tong University School of Medicine, Shanghai, China; ^5^Center for Community Health Care, Hospital Development Institute Shanghai Jiao Tong University, Shanghai, China

**Keywords:** transgender women, childhood sexual experiences, intimate partner violence, suicide, regression

## Abstract

**Objective:**

Several studies highlighted childhood sexual experiences (CSEs) and intimate partner violence (IPV) as risk factors that affected lifetime suicidal ideation. TW had higher rates of CSEs and IPV than cisgender people. The aim of this study was to comprehensively assess the prevalence of CSEs and IPV among TW and their association with lifetime suicidal ideation.

**Methods:**

A cross-sectional survey was conducted among 247 TW in Shenyang and Kunming, China, from April to September 2018. CSEs, IPV, and lifetime suicidal ideation were assessed. Logistic regression models were used to examine the association between self-reported CSEs under 18 years of age, IPV in adulthood, and lifetime suicidal ideation.

**Results:**

In the study, 14.2% (35/247) of the sample participants reported CSEs under 18 years of age; 44.9% (111/247) reported experiencing IPV in adulthood, including 18.6% (44/247) of physical IPV, 27.1% (67/247) of trans-specific identity IPV, 31.6% (78/247) of verbal IPV, and 19.4% (48/247) of sexual IPV; and 26.3% (65/247) had thought about attempting suicide at least one time. CSEs and any form of IPV were significantly associated with suicidal ideation in this sample population. A final stepwise multivariate logistic regression model found that both physical and verbal IPVs were significantly associated with suicidal ideation when controlling for other factors (ORm_1_ = 2.58, 95% confidence interval (CI) = 1.163–5.724; ORm_2_ = 2.72, 95% CI = 1.334–5.547).

**Conclusions:**

The findings highlight the effects of CSEs and IPV among TW and suggest the need for research on suicide in the future. Suicide prevention efforts for this invisible and vulnerable population should focus on those with physical and verbal IPV.

## Introduction

Transgender women (TW) are a group of people belonging to the transgender population, for whom the assigned sex at birth is male but they identify as female (1). As a marginalized group, TW experience negative health outcomes due to their minority group status, such as anxiety, depression, and even suicide (2); however, TW are routinely overlooked in public health research (3). The prevalence of suicidal ideation among the transgender population is extremely high in developed countries, such as Canada, some European countries, and the USA. The rate of lifetime suicidal ideation ranges from 25 to 48% (4–6). A cross-sectional study in China found that TW had a high rate of suicidal ideation or a suicide attempt (22% reported only suicidal ideation and 25.6% reported a prior suicide attempt) (7).

A systematic review found that young sexual minorities were more than three times more likely to attempt suicide than their heterosexual counterparts, and transgender people were nearly six times as likely (8). Compared with transgender men, TW's less favorable societal position is associated with a greater risk for suicide behaviors (9). A recent Chinese study among the transgender population found that the suicide risk among TW (72.87%) was higher than that among transgender men (54.93%) (10). In China, imperfect transgender medical treatment and a lack of relevant laws and regulations are major obstacles to successfully dealing with identifying oneself as a transgender individual. Moreover, due to the limitations of traditional ideas in Chinese culture, Chinese parents are often unable to accept transgender children, which enables us to assume that Chinese transgender individuals often do not have sufficient family and social support, possibly contributing to a high risk of suicide (11).

Intimate partner violence (IPV) was considered an essential risk factor for suicidal ideation among transgender people. A recent study analyzed suicide behaviors among African American transgender individuals and found that individuals who had experienced IPV were at the highest risk for a suicide attempt (4). Previous research examining the lifetime prevalence of IPV and its association with health among transgender people aged 50 and older found that 57% of participants reported any lifetime IPV, and their IPV experience was significantly associated with suicide (12). In a multisite cohort of young TW in the USA, the lifetime prevalence of IPV was 42% (13).

However, the lifetime prevalence of IPV among Chinese transgender people remains unclear because nationally representative data are even less available. The finding of a study examining the prevalence and risk factors of suicidal ideation among TW in China was that many TW experienced discrimination, including verbal abuse (56%), physical abuse, and sexual violence (32%) in their lifetimes, which was also significantly associated with suicidal ideation or a prior suicide attempt (7). A recent review of the lifetime experience of IPV among transgender people aged 18 and older, based on more than 85 published studies and previously unanalyzed data sets involving partner abuse, reported a wide range of estimates, from 8 to 73% for trans-specific, 9 to 83% for psychological, 10 to 67% for physical, 5 to 67% for sexual, and 6 to 83% for any IPV (14).

Additionally, existing studies found associations between early sexual experiences, high-risk sexual behaviors, and a higher likelihood of suicidality (15–18). Numerous studies reported differences in childhood sexual experiences (CSEs) across racial and ethnic groups, with higher rates among the minority groups in the USA (19). An interview-based study was conducted to assess the prevalence of CSEs with an older partner among men who had sex with men (MSM) and transgender people in Campinas, Brazil, of which 32% reported CSEs with an older partner; transgender people were significantly more likely to report such experiences, and compared with men, they had reduced negative feelings about their experiences at the time of the interview (20). Several studies estimated that 50–61% of TW have CSEs (21, 22). However, the prevalence of CSEs among Chinese TW is not available. Studies in China investigating MSM showed that 26.5–31.51% reported to have experienced CSEs (23, 24), which was greater than that reported in a meta-analysis of 27 studies in China; the prevalence of CSEs was 13.8% in boys and 15.3% in girls (25).

Although many previous studies investigated suicide-related conditions and clinical populations (26–28), studies examining the suicide risk in TW populations in China is lacking. Therefore, in this present exploratory study, we explored the CSEs and IPV among TW, including their potential associations with lifetime suicidal ideation. Based on previous findings in the literature, the following hypotheses were generated: (a) CSEs and IPV would be reported among TW, and (b) CSEs and IPV would be associated with the reported lifetime suicidal ideation.

## Methods

### Study design and subjects

A cross-sectional survey was conducted in Shenyang and Kunming, China, from April to September 2018. A snowball sampling method was used for recruitment with the help of a non-governmental organization (NGO). The staff of the NGO who provided services to TW contacted all their clients and performed outreach for recruitment. With the assistance of the NGO, five eligible TW were recruited as “seeds.” These “seeds” then each recruited or recommended other suitable ones; all participants forwarded the questionnaire survey in this way until saturation was met (the recruited participants could no longer introduce new participants). The saturation was also double-checked by NGO leaders, who worked with local TW and have an approximate number of TW living locally. In this way, all participants forwarded the questionnaire survey until they were unable to identify others who met the inclusion criteria. The inclusion criteria were as follows: (1) TW who are 18 years of age or older; (2) those who were male sex assigned at birth; (3) those who self-identified as being female; and (4) those who understood the study procedures and provided written informed consent.

All participants were guaranteed anonymity and given the right to withdraw at any time with no consequences for refusal to participate in the survey. A total of 247 TW were ultimately recruited for the study. Participants' ages ranged from 18 to 61 years, with a mean age of 33.04 years.

### Ethical statement

Ethical approval for the study was provided by the Public Health and Nursing Ethics Committee of Shanghai Jiao Tong University School of Medicine. We obtained consent from all participants online before the survey commenced.

## Measures

### Childhood sexual experiences

One yes/no item: “Before you turned 18, did a person threaten and force you to submit to sex acts?” Coded 0 = no if the respondent answered “no” or 1 = yes if the respondent answered “yes.”

### Intimate partner violence

Intimate partner violence was a simplified questionnaire that was developed from the Transgender Youth Research Project (29), and four items were included: “Have any of your romantic or sexual partner ever…” to identify physical, verbal, sexual, and trans-specific identity IPV. Coded 0 = no if participants responded “no” or 1 = yes if participants responded “yes” to either item. In the present study, the internal consistency reliability of IPV was 0.767.

### Lifetime suicidal ideation

One yes/no question: “At any time in your life, have you ever considered suicide?” Coded 0 = no if the respondent answered “no” or 1 = yes if the respondent answered “yes.”

### Statistical analysis

All data were recorded and processed using SPSS Statistics 28.0. Quantitative variables were reported as mean ± standard deviation (SD), and qualitative variables were expressed as numbers (percentages). The correlation analysis was assessed with Spearman's rank correlation test. To examine if independent variables (e.g., sexual orientation, the subtypes of IPV, and CSEs) affect suicidal ideation, collinearity diagnostics were performed by using tolerance (Tol) and variance inflation factor (VIF) to evaluate multi-collinearity, with a Tol of <0.1 or a VIF of >10 indicating multi-collinearity. A multivariate logistic analysis was performed using the likelihood ratio (LR) method to analyze suicidal ideation as an outcome with CSEs and subtypes of IPV as predictors. Models were controlled for sexual orientation. The level of significance was considered to be < 0.05 (*p* < 0.05).

## Results

Table 1 presents the demographic characteristics and prevalence of CSEs and each IPV. This sample population was mostly under the age of 40 years (83.8%), had a homosexual orientation (56.3%), had senior and lower education (68.8%), was single (66.8%), and had 6,000 RMB and a lower income (71.6%). Those who reported ever thinking about attempting suicide were 26.3%. Univariate logistic regression showed that sexual orientation had a significant association with suicidal ideation. Compared to those with heterosexual and sexual orientation, those with unknown sexual orientation had higher rates of suicidal ideation (odds ratio (OR) = 5.564, 95% confidence interval (CI) = 1.753–17.661).

**Table 1:** Demographic characteristics of transgender women (TW) and association with suicidal ideation (*n* = 247).

**Characteristic**	***N* (%)**	**Suicidal ideation**	
		***N*** **(%)**	**ORu (95%CI)**
**Age (year)**			
18–25	42 (17.0)	12 (28.6)	1
25–40	165 (66.8)	42 (25.5)	0.854 (0.401–1.817)
41–61	40 (16.2)	11 (27.5)	0.948 (0.362–2.487)
**Sexual orientation**			
Heterosexual	37 (15.0)	6 (16.2)	1
Homosexual	139 (56.3)	36 (25.9)	1.806 (0.696–4.683)
Bisexual	44 (17.8)	9 (20.5)	1.329 (0.425–4.156)
Unknown	27 (10.9)	14 (51.9)	5.564 (1.753–17.661)^**^
**Education**			
Primary and lower	20 (8.1)	5 (25.0)	1
Junior	70 (28.3)	19 (27.1)	1.118 (0.357–3.498)
Senior	80 (32.4)	18 (22.5)	0.871 (0.279–2.724)
College and higher	77 (31.2)	23 (29.9)	1.278 (0.415–3.930)
**Marital status**			
Single	201 (81.4)	55 (27.4)	1
Married	17 (6.9)	5 (29.4)	1.106 (0.372–3.284)
Divorced or widowed	29 (11.7)	5 (17.2)	0.553 (0.201–1.522)
**Income**			
≤ ¥3,000	72 (29.1)	21 (29.2)	1
¥3,001–¥6,000	105 (42.5)	29 (27.6)	0.927 (0.477–1.801)
≥¥6,001	70 (28.3)	15 (21.4)	0.662 (0.308–1.422)
**Residence**			
Local	93 (37.7)	45 (29.2)	1
Not local	154 (62.3)	20 (21.5)	1.507 (0.823–2.758)
**Lifetime suicidal ideation**	65 (26.3)	–	–

ORu, odds ratio in univariate logistic regression; 95% CI, 95% confidence interval;

** *p* < 0.01.

Table 2 presents the correlation analysis of CSEs, IPV, and lifetime suicidal ideation. Suicidal ideation was found to be significantly associated with CSEs and each IPV. Overall, 14.2% experienced at least CSEs, with 44.9% reporting any kind of IPV. Within IPV, verbal IPV was the most prevalent (31.6%) and physical IPV was the least prevalent (18.6%) (see Table 3).

**Table 2:** Correlation analysis of CSEs, and of IPV, and suicidal ideation, spearman's rank correlation test.

**Variable**	**1**	**2**	**3**	**4**	**5**	**6**
1. Suicidal ideation	1					
2. CSEs	0.153^*^	1				
3. Physical IPV	0.281^***^	0.223^***^	1			
4. Verbal IPV	0.286^***^	0.273^***^	0.247^***^	1		
5. Sexual IPV	0.148^*^	0.299^***^	0.448^***^	0.459^***^	1	
6. Trans-specific identity IPV	0.256^***^	0.327^***^	0.386^***^	0.506^***^	0.414^***^	1
7. IPV	0.236^***^	0.263^***^	0.530^***^	0.752^***^	0.544^***^	0.675^***^

CSEs, childhood sexual experiences; IPV, intimate partner violence;

* *p* < 0.05,

*** *p* < 0.001.

**Table 3:** Multiple liner regression analysis with collinearity diagnostics.

**Variable**		***N* (%)**	**Suicidal ideation**					
			***N*** **(%)**	**ORu** **(95% CI)**	**AOR** **(95% CI)**	**ORm** **(95% CI)**	**Tol**	**VIF**
CSEs	No#	212 (85.8)	50 (23.6)	1	1		0.848	1.179
	Be	35 (14.2)	15 (42.9)	2.430 (1.159–5.097)^*^	3.023 (1.402–6.521)^**^			
Physical IPV	No#	201 (81.4)	41 (20.4)	1	1		0.675	1.482
	Be	46 (18.6)	24 (52.2)	4.257 (2.173–8.341)^***^	4.493 (2.244–8.994)^***^	2.58 (1.163–5.724)^*^		
Verbal IPV	No#	169 (68.4)	30 (17.8)	1	1		0.601	1.663
	Be	78 (31.6)	35 (44.9)	3.771 (2.079–6.842)^***^	4.043 (2.172–7.525)^***^	2.72 (1.334–5.547)^**^		
Sexual IPV	No#	199 (80.6)	46 (23.1)	1	1		0.682	1.466
	Be	48 (19.4)	19 (39.6)	2.179 (1.120–4.241)^*^	2.334 (1.173–4.644)^*^			
Trans-specific identity IPV	No#	180 (72.9)	35 (19.4)	1	1		0.667	1.499
	Be	67 (27.1)	30 (44.8)	3.359 (1.831–6.162)^***^	3.887 (2.054–7.356)^***^			

AOR, adjusted odds ratio in univariate logistic regression, models adjusted for sexual orientation; ORm, odds ratio in stepwise multivariate logistic regression, models adjusted for sexual orientation; #Control group; Tol, tolerance, VIF, variance inflation factor.

Estimates of odds ratio from logistic regression models for suicidal ideation

* *p* < 0.05,

** *p* < 0.01,

*** *p* < 0.001.

As shown in Table 3, the multilinear regression analysis with collinearity diagnostics detected no collinearity among CSEs and the subtypes of IPV of participants (all Tol > 0.1, all VIF < 10). Any forms of IPV and CSEs were included in the univariate logistic regression model after correcting sexual orientation. Table 3 presents a significant association of all factors with suicidal ideation through univariate logistic regression (*ps* < 0.05). Next, we conducted a stepwise multivariate logistic regression, CSEs, IPV, physical IPV, verbal IPV, sexual IPV, and trans-specific identity IPV which were included in the analysis at one time. After adjusting for sexual orientation, the results demonstrated a general increase in the likelihood of reporting suicidal ideation as IPV ever experienced physical and verbal IPV. This relationship was statistically significant for suicidal ideation. Participants who reported experiencing physical and verbal IPV had about three times the odds of reporting suicidal ideation (ORm_1_ = 2.58, 95% CI = 1.163–5.724; ORm_2_ = 2.72, 95%CI = 1.334–5.547).

## Discussion

This exploratory study provides initial findings to understand the impact of Chinese TW on their CSEs, IPV, and the lifetime prevalence of suicidal ideation. The results revealed that the rate of TW who reported that they ever thought of attempting suicide was 26.3%, which was similar to previous research findings (30–32). The estimated prevalence among TW is greater than that among the general Chinese population, where 12–16% experienced suicidal ideation and 2–3% had attempted suicide (30, 33, 34). Respondents who had ever experienced CSEs and IPV were significantly more likely to view their current general health negatively and to have ever thought about suicide (12, 35). Moreover, we found that participants who reported experiencing physical and verbal IPV had about three times more odds of reporting suicidal ideation using a stepwise logistic regression.

Overall, CSEs were experienced in this sample of TW (14.2%) and were strongly associated with suicidal ideation. In accordance with previous research from other developed countries (20), the low rate of CSEs among TW in China was reported. Given the findings that suggest that transgender people in China are less likely to have access to healthcare and lack adequate legal protections (36), reporting sexual experiences without biasing responses and overcoming the natural resistance to disclose a stigmatized behavior further lowers the rate of CSEs among TW (37, 38). However, multivariate logistic analysis in the present study could not find that the participants exposed to CSEs had about the same odds of reporting suicidality as those exposed to no CSEs, which is different from previous studies on adverse childhood experiences and suicidal ideation (35, 39, 40). There could be any number of reasons this could happen; we proposed that there was a more dominant variable behind CSEs, namely, the individual's perceived burdensomeness, thwarted belongingness, and social isolation. According to the interpersonal theory of suicide (41), children who were abused might develop a desire for suicide from a sense of isolation, shame, and self-hatred and suggest that they were a burden on their family or that their family would be better off if they were dead. Therefore, there are reasons to assume that some variables (e.g., interpersonal needs) may act as a mediator between CSEs and suicidal ideation (42).

Consistent with predictions from the Gender Minority Stress Framework (43), in which TW might face synergistic and unique forms of abuse due to their transgender status, 44.9% were subjected to any kind of IPV from their partners or customers in their lifetime. Verbal IPV was the most prevalent (31.6%) and physical IPV was the least prevalent (18.6%), consistent with reports of the lifetime prevalence of IPV in the literature among transgender adults (31–50%) (44, 45). The prevalence of IPV is far higher in the current year than in the past few years. For example, a study explored the past year prevalence of IPV, finding that TW (past year prevalence of 12.1%) experienced one to two times higher odds of physical or sexual IPV than the subgroups of transgender and gender non-conforming individuals (the past year prevalence of 6.6–9.1%), and six times higher odds than cisgender women (the past year prevalence of 2.7%). The fact is most likely due to TW having a well-documented risk for IPV (46).

The high prevalence of IPV may substantially contribute to the health problems experienced by TW. In the current research, participants exposed to physical and verbal IPV had about three times the odds of reporting suicidality than those exposed to no IPV. Our findings echo those of Hillman et al. (12), who demonstrated that 57% of respondents reporting any lifetime IPV experience rather than no IPV experience was significantly associated with a suicide attempt. Previous research linked victimization to several pernicious health outcomes, including depression, anxiety, and elevated rates of suicide (47). As one is victimized repeatedly, suicide may be an “escape” from that victimization.

Participants exposed to sexual and trans-specific identity IPV did report more suicidal ideation. The TW group itself can give a reasonable explanation: commercial sex is one of the most important sources of income for many TW, and a considerable number of TW take it as their permanent career (48). Meanwhile, commercial sex reinforces their female gender identity (49). In addition, these two IPVs appear through physical and verbal IPV among TW, which lead to non-significant results. Although some studies showed that both IPV and CSEs are associated with depression (12, 35), anxiety (50, 51), post-traumatic stress disorder (PTSD) (35, 52), etc., which are related to suicide (7, 10, 30, 53), few studies explored whether these factors mediate the pathway from IPV/CSEs to suicidal ideation. Future research is needed to focus on the processes or mechanisms among these factors, especially among TW in China. Prevention strategies (e.g., creating protective environments, teaching skills to prevent physical and verbal violence, and providing opportunities to create empowered women) (54) may prove effective in reducing victimization rates among Chinese TW and thus may also help lessen suicide tendencies.

Several limitations in our study should be noted. First, because the data were cross-sectional and most variables were measured using self-designed single-item questions, in particular, CSEs' onset age, duration, frequency, and severity over time were not available, and the relationships between CSEs and suicidal ideation should be interpreted with caution; in the future, well established scales need to be chosen. In addition, self-reporting of CSEs that occurred in the past few years might be subject to recall and self-disclosure biases. Similarly, no information was available about IPV in adulthood; future research in another direction might be taken into account. Second, the sample size was relatively small. Third, adopting snowball sampling in Shenyang and Kunming implied that the applicability of the findings to other areas in China might be limited. Future research is needed to cover the diversity of TW across China.

## Conclusion

This present study found that physical and verbal IPV in adulthood were prevalent among TW, which was significantly related to lifetime suicidal ideation. Prevention and intervention strategies should be aimed at reducing IPV and suicide attempts, especially for those who had ever experienced physical and verbal IPV among TW.

## Data availability statement

The original contributions presented in the study are included in the article/supplementary material, further inquiries can be directed to the corresponding authors.

## Ethics statement

The studies involving human participants were reviewed and approved by Public Health and Nursing Ethics Committee of Shanghai Jiao Tong University School of Medicine. Written informed consent to participate in this study was provided by the participants' legal guardian/next of kin.

## Author contributions

LX and RC analyzed the data and wrote the manuscript. YC, DX, CX, XY, HC, RW, YL, SL, XG, TM, and YW participated in the experimental design and data collection. YZ and SM gave the guidance for manuscript revision. SM and YC gave guidance on the experimental design and manuscript writing. All authors contributed to the whole process of the research.
